# Sleep quality, daytime sleepiness, fatigue, and quality of life in patients with multiple sclerosis treated with interferon beta-1b: results from a prospective observational cohort study

**DOI:** 10.1186/s12883-018-1113-5

**Published:** 2018-08-24

**Authors:** Sylvia Kotterba, Thomas Neusser, Christiane Norenberg, Patrick Bussfeld, Thomas Glaser, Martin Dörner, Markus Schürks

**Affiliations:** 1 Klinik für Geriatrie, Klinikum Leer gemeinnützige GmbH, Leer, Germany; 2 Bayer Vital GmbH, Leverkusen, Germany; 3 Bayer AG, Wuppertal, Germany; 4 Bayer Consumer Care AG, Basel, Switzerland

**Keywords:** Multiple sclerosis, Interferon beta-1b, Sleep quality, Fatigue, Functional health status, Real world

## Abstract

**Background:**

Sleep disorders and fatigue are common in multiple sclerosis (MS). The underlying causes are not fully understood, and prospective studies are lacking. Therefore, we conducted a prospective, observational cohort study investigating sleep quality, fatigue, quality of life, and comorbidities in patients with MS.

**Methods:**

Patients with relapsing-remitting MS or clinically isolated syndrome treated with interferon beta-1b were followed over two years. The primary objective was to investigate correlations between sleep quality (PSQI), fatigue (MFIS), and functional health status (SF-36). Secondary objectives were to investigate correlations of sleep quality and daytime sleepiness (ESS), depression (HADS-D), anxiety (HADS-A), pain (HSAL), and restless legs syndrome (RLS). We applied descriptive statistics, correlation and regression analyses.

**Results:**

139 patients were enrolled, 128 were available for full analysis. The proportion of poor sleepers (PSQI≥5) was 55.47% at the beginning and 37.70% by the end of the study (106 and 41 evaluable questionnaires, respectively). Poor sleepers performed worse in MFIS, SF-36, ESS, HADS-D, and HADS-A scores. The prevalence of patients with RLS was low (4.5%) and all were poor sleepers. Poor sleep quality was positively correlated with fatigue and low functional health status. These relationships were corroborated by multivariable-adjusted regression analyses. ESS values and poor sleep quality at baseline seem to predict sleep quality at the one-year follow-up. No variable predicted sleep quality at the two-year follow-up.

**Conclusions:**

Our results confirm the high prevalence of poor sleep quality among patients with MS and its persistent correlation with fatigue and reduced quality of life over time. They highlight the importance of interventions to improve sleep quality.

**Trial registration:**

The study was registered at clinicaltrials.gov: NCT01766063 (registered December 7, 2012). Registered retrospectively (first patient enrolled December 6, 2012).

**Electronic supplementary material:**

The online version of this article (10.1186/s12883-018-1113-5) contains supplementary material, which is available to authorized users.

## Background

Multiple sclerosis (MS) is a chronic inflammatory and degenerative autoimmune disorder affecting more than two million people worldwide [1]. The prevalence is higher in women than in men. MS is a frequent cause of nontraumatic neurological disability in young adults [1].

Comorbid conditions are common in MS and may contribute to disability. Many patients with MS report sleep disorders [2], more frequently than in the general population, with prevalence estimates ranging from 25 to 54% [3]. Poor sleep quality in MS has been associated with negative outcomes, such as decreased quality of life [4], exacerbation rate and disease severity [5], and with other comorbidities such as fatigue, depression, anxiety, and pain [6, 7].

Fatigue is another common symptom in patients with MS and is closely connected with sleep disorders [3, 8]. Treatment of sleep disorders may have the potential to improve fatigue [9–11].

The underlying causes of poor sleep quality and fatigue are not fully understood. Restless legs syndrome (RLS) appears to play an important role since it has consistently been shown to be more common in patients with MS [2, 9, 12] and is associated with poor sleep [8, 13, 14]. The type of MS treatment may also impact sleep and fatigue. Disease-modifying drugs (DMD), such as interferon beta-1b, might affect sleep quality and fatigue, but results in this connection are ambiguous [8, 13, 14].

Available studies are small and have included cohorts of patients on various treatments. Prospective studies on sleep quality and fatigue are lacking.

Hence, we conducted a prospective study investigating sleep quality, fatigue, quality of life, and comorbidities in patients with MS in a real-world setting over the course of two years. In order to exclude influence of various disease modifying drugs, only patients with interferon beta-1b were included.

## Methods

### Study design

The BETASLEEP study (NCT01766063) was a prospective, observational cohort study in Germany sponsored by Bayer Vital GmbH. Patients were recruited from 35 neurological offices and clinics specializing in the treatment of MS between December 2012 and January 2015. Patients were followed up for a total of 24 months, with documented visits at baseline, 6, 12, 18, and 24 months. Detailed information about the data collection process and training of investigators is provided in the Additional file 1.

### Eligibility

Eligible patients had relapsing-remitting MS (RRMS) or clinically isolated syndrome (CIS), were at least 18 years old, and had an EDSS (expanded disability status scale) score ≤ 5. Furthermore, patients had to be on treatment with interferon beta-1b. Treatment duration was to be not more than six months and the treatment had to be tolerated by the patient according to their attending physician. All patients provided their written informed consent to participate in the study.

### Objectives

Primary objectives were to investigate correlations between sleep quality, fatigue, and functional health status. Secondary objectives were to investigate correlations of sleep quality and daytime sleepiness, depression, anxiety, pain, and RLS.

### Outcome variables

Primary outcome variables were sleep quality assessed with the Pittsburgh Sleep Quality Index (PSQI), fatigue assessed with the Modified Fatigue Impact Scale (MFIS), and functional health status assessed with the Short Form 36 (SF-36). Secondary outcome variables were daytime sleepiness measured with the Epworth Sleepiness Scale (ESS), depression and anxiety assessed with the Hospital Anxiety and Depression Scale (HADS), pain measured with the Hamburg Pain Adjective List (HSAL, Hamburger Schmerz Adjektiv Liste), and the severity of RLS assessed through the International RLS Study Group (IRLSSG) rating scale. Detailed information about the questionnaires is provided in the Additional file 1.

### Statistical analyses

Statistical analyses were performed using SAS version 9.4 (SAS Institute Inc., Cary, NC). All analyses were exploratory. Continuous variables were described by sample statistics and categorical variables by frequency tables displaying the number of patients as well as percentages. The analyses were performed for the total population and stratified by baseline PSQI score (< 5, ≥5).

Correlations between the primary outcome variables and between sleep quality and the secondary outcome variables were calculated using Spearman rank correlation. Analyses were performed at baseline and all follow-up visits.

To further investigate the impact of potential confounders on the correlations, we also performed multivariable-adjusted regression analyses at baseline controlling for age, gender, EDSS score, and duration of disease.

In order to determine potential baseline predictors of poor sleep quality (PSQI≥5) at 12 and 24 months, we first performed univariate logistic regression for the dependent variable (PSQI< 5 vs. PSQI≥5). Second, we employed a stepwise selection procedure with an entry level of *p* = 0.5 and a stay level of *p* = 0.1. The following independent covariates were considered: gender (female, male), age (years), BMI (kg/m^2^), type of MS (CIS, RRMS), baseline EDSS score (< 3, ≥3), baseline PSQI score (< 5, ≥5), MS duration (months), duration of interferon beta-1b treatment (< 3 months, ≥3 months), previous sleep disorder (no, yes), baseline ESS score, baseline HADS depression and anxiety scores (< 8, ≥8), and concomitant medication (no, yes) until initial visit.

For primary outcome variables, missing data were not imputed. Questionnaires were scored according to standard rules based on available instructions. For the regression models in secondary analyses, missing values in the questionnaire scores were either replaced by the mean or median of the available values (continuous data) or a separate category was created (categorical data).

In order to account for decreasing sample size, we performed sensitivity analyses among patients with available data at each visit.

## Results

### Patient disposition

From December 2012 to January 2015 a total of 139 patients were enrolled into the study, 128 patients were available for full analysis. A flow chart describing patient disposition is provided in Additional file 2. 45.5% of all patients completed the study. Of the patients who discontinued participation in the study, 35.3% were lost to follow up, 23.5% withdrew consent to participate in the study, 13.7% switched to another medication, and 27.5% discontinued study participation for other reasons.

### Baseline characteristics

Baseline characteristics are summarized in Table 1. The median age of the sample was 41 years (range 19–70 years; mean 41.5; SD = 11.3), and 71.1% were female. 89.1% had RRMS, while 10.9% had CIS. The median duration of disease was 6.9 months (range 0.1–315.1 months). The median EDSS was 2 (range 0–5). Some differences in gender and disease duration were seen between the good and the poor sleepers.

**Table 1:** Baseline characteristics and scores

Characteristic	All Patients	Good sleepers (PSQI< 5)	Poor sleepers (PSQI≥5)
Age, years	*N* = 128	*N* = 35	*N* = 71
Mean (SD)	41.5 (11.3)	40.4 (11.8)	41.3 (10.7)
Median (range)	41.0 (19–70)	41.0 (19–61)	41.0 (19–65)
Gender, n (%)	*N* = 128	*N* = 35	*N* = 71
Women	91 (71.1)	21 (60.0)	53 (74.7)
Men	37 (28.9)	14 (40.0)	18 (25.4)
Diagnosis, n (%)	*N* = 128	*N* = 35	*N* = 71
RRMS	114 (89.1)	31 (88.6)	62 (87.3)
CIS	14 (10.9)	4 (11.4)	9 (12.7)
Duration of disease, months	*N* = 113	*N* = 32	*N* = 61
Mean (SD)	43.0 (71.6)	30.9 (63.8)	45.6 (74.3)
Median (range)	6.9 (0.1–315.1)	6.9 (0.3–262.3)	6.3 (0.1–315.1)
EDSS, median (range)	*N* = 128	*N* = 35	*N* = 71
	2.0 (0–5)	2.0 (0–5)	2.0 (0–5)
MFIS	*N* = 122	*N* = 35	*N* = 71
Mean (SD)	32.38 (20.33)	20.20 (15.24)	39.38 (18.78)
Median (range)	34.0 (0.0–76.0)	18.0 (0.0–51.0)	43.0 (2.0–76.0)
SF-36 physical component score	*N* = 113	*N* = 33	*N* = 67
Mean (SD)	44.56 (11.35)	50.86 (8.37)	41.80 (11.41)
Median (range)	46.53 (16.50–64.06)	53.00 (22.67–64.06)	41.21 (16.50–60.67)
SF-36 mental component score	*N* = 113	*N* = 33	*N* = 67
Mean (SD)	41.74 (13.28)	47.84 (9.98)	38.27 (13.31)
Median (range)	44.39 (12.44–63.82)	50.96 (22.76–63.82)	39.54 (12.44–59.53)
HADS-D	*N* = 128	*N* = 35	*N* = 71
HADS-D ≥ 8, n (%)	29 (22.66)	2 (5.71)	25 (35.21)
HADS-A	*N* = 128	*N* = 35	*N* = 71
HADS-A ≥ 8, n (%)	41 (32.03)	4 (11.43)	32 (45.07)
ESS	*N* = 118	*N* = 35	*N* = 68
Mean (SD)	8.03 (4.54)	6.69 (4.26)	8.88 (4.71)
Median (range)	8.0 (0.0–16.0)	6.0 (0.0–14.0)	9.0 (1.0–16.0)

Subgroups of good sleepers (PSQI< 5) and poor sleepers (PSQI≥5) do not add up to *N* = 128 (100%) due to missing PSQI baseline values

*PSQI* Pittsburgh Sleep Quality Index, *SD* standard deviation, *RRMS* relapsing-remitting multiple sclerosis, *CIS* clinically isolated syndrome, *EDSS* Expanded Disability Status Scale, *MFIS* Modified Fatigue Impact Scale, *SF-36* Short Form 36, *HADS* Hospital Anxiety and Depression Scale, *ESS* Epworth Sleepiness Scale

### Sleep quality

At the initial visit, the mean PSQI score was 7.31 (SD = 4.36; median 6; range 1–18). Among 128 patients (106 patients had evaluable PSQI questionnaires) 55.47% indicated poor sleep quality (Table 2, Additional file 3). The mean and median PSQI scores at the final visit were lower (mean 6.71; SD = 4.11; median 5; range 1–18), with 37.70% of 61 patients (41 patients with evaluable PSQI questionnaires) indicating poor sleep quality.

**Table 2:** Course of sleep quality throughout the study

Patients	Baseline visit	6-month visit	12-month visit	18-month visit	24-month visit
All patients, N	128	109	96	65	61
Patients with evaluable questionnaires, N	106	90	82	51	41
PSQI mean (SD)	7.31 (4.36)	6.37 (3.99)	6.43 (4.03)	6.45 (4.48)	6.71 (4.11)
PSQI median (range)	6.0 (1.0–18.0)	5.5 (1.0–20.0)	5.0 (1.0–18.0)	5.0 (0.0–18.0)	5.0 (1.0–18.0)
Proportion of patients with PSQI≥5 (95% confidence interval)	55.47 (46.43–64.25)	46.79 (37.17–56.59)	50.00 (39.62–60.38)	49.23 (36.60–61.93)	37.70 (25.61–51.04)
Sensitivity analysis, N	28	28	28	28	28
PSQI mean (SD)	6.75 (3.95)	6.43 (4.26)	6.00 (3.22)	6.21 (4.07)	6.29 (3.61)
PSQI median (range)	5.0 (1.0–14.0)	4.0 (1.0–18.0)	5.5 (2.0–14.0)	5.0 (1.0–18.0)	5.0 (1.0–16.0)
Proportion of patients with PSQI≥5 (95% confidence interval)	57.14 (37.18–75.54)	46.43 (27.51–66.13)	57.14 (37.18–75.54)	64.29 (44.07–81.36)	53.57 (33.87–72.49)

*PSQI* Pittsburgh Sleep Quality Index, *SD* standard deviation

In the sensitivity analysis considering only patients with PSQI scores at all visits (*n* = 28), the mean PSQI score at baseline was 6.75 (SD = 3.95; median 5; range 1–14), and 57.14% of patients indicated poor sleep quality. At the final visit, the mean PSQI was 6.29 (SD = 3.61; median 5; range 1–16), and 53.57% of patients indicated poor sleep quality.

### Health status course

**Fig. 1:**
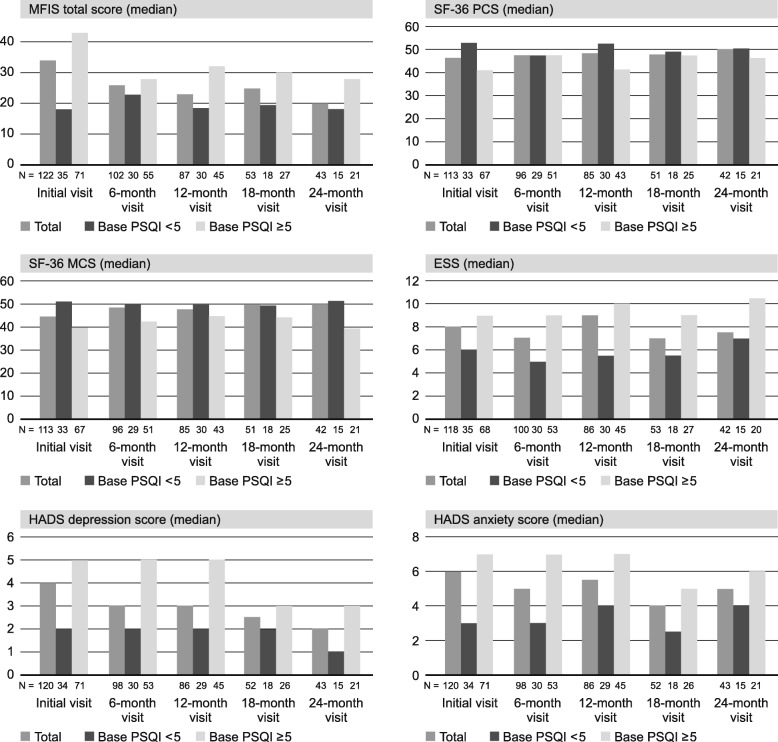
Course of fatigue (MFIS), functional health (SF-36), sleepiness (ESS), depression, and anxiety (HADS) throughout the study. Numbers of good sleepers (PSQI< 5) and poor sleepers (PSQI≥5) do not add up to number of all patients due to missing PSQI baseline values *MFIS* Modified Fatigue Impact Scale, *SF-36* Short Form-36, *PCS* physical component score, *MCS* mental component score, *ESS* Epworth Sleepiness Scale, *HADS* Hospital Anxiety and Depression Scale.

At the initial visit, the mean MFIS score was 32.4 (SD = 20.3; median 34; range 0–76; Fig. 1). Poor sleepers had a higher MFIS score (mean 39.4; SD = 18.8; median 43; range 2–76) than good sleepers (mean 20.2; SD = 15.2; median 18; range 0–51). The differences between poor and good sleepers were apparent at each visit. The sensitivity analysis among participants with available data at each visit confirmed these findings.

Poor sleepers also performed worse than good sleepers in the mean SF-36 physical (PCS) and mental component scores (MCS; Fig. 1). These differences could be observed at each visit. In the sensitivity analysis, the differences between poor sleepers and good sleepers in the PCS were less pronounced, while the differences in the MCS were confirmed.

With respect to the ESS, HADS depression, and HADS anxiety scores, poor sleepers performed worse at each visit (Fig. 1).

The prevalence of RLS in the sample was low (4.48% [*n* = 6] at initial visit, 6.56% [*n* = 4] at final visit); all patients diagnosed with RLS were poor sleepers (Table 3). Likewise, the number of patients with reported chronic pain was low, hence the low number of HSAL scores (Table 3).

**Table 3:** Course of symptom severity, restless legs syndrome, and pain throughout the BETASLEEP study

Characteristic	Baseline visit			6-month visit			12-month visit			18-month visit			24-month visit		
	All Patients	Good sleepers (PSQI< 5)	Poor sleepers (PSQI≥5)	All Patients	Good sleepers (PSQI< 5)	Poor sleepers (PSQI≥5)	All Patients	Good sleepers (PSQI< 5)	Poor sleepers (PSQI≥5)	All Patients	Good sleepers (PSQI< 5)	Poor sleepers (PSQI≥5)	All Patients	Good sleepers (PSQI< 5)	Poor sleepers (PSQI≥5)
EDSS	*N* = 128	*N* = 35	*N* = 71	*N* = 90	*N* = 26	*N* = 50	*N* = 75	*N* = 25	*N* = 40	*N* = 50	*N* = 16	*N* = 27	*N* = 49	*N* = 17	*N* = 25
Median (range)	2.0 (0.0–5.0)	2.0 (0.0–5.0)	2.0 (0.0–5.0)	2.0 (0.0–6.5)	2.0 (0.0–4.0)	2.0 (0.0–6.5)	2.0 (0.0–6.5)	1.5 (0.0–4.5)	2.0 (0.0–6.5)	2.0 (0.0–6.5)	1.5 (0.0–4.5)	2.0 (0.0–6.5)	2.0 (0.0–5.0)	1.5 (0.0–5.0)	2.0 (0.0–5.0)
MSFC	*N* = 93	*N* = 26	*N* = 55				*N* = 54	*N* = 18	*N* = 31				*N* = 31	*N* = 8	*N* = 17
Median (range)	0.11 (− 3.46–1.49)	0.21 (− 1.01–0.69)	0.08 (− 3.46–1.49)				0.20 (− 1.13–0.87)	0.26 (− 0.76–0.87)	0.22 (− 1.13–0.80)				0.17 (− 1.52–0.99)	0.20 (− 0.46–0.73)	0.17 (− 1.52–0.99)
RLS, evaluable patients (N)	*N* = 128	*N* = 35	*N* = 67	*N* = 109	*N* = 33	*N* = 59	*N* = 96	*N* = 31	*N* = 50	*N* = 65	*N* = 20	*N* = 34	*N* = 61	*N* = 20	*N* = 31
n (%)	5 (3.9)	0	4 (6.0)	3 (2.8)	0	2 (3.4)	4 (4.2)	0	2 (4.0)	1 (1.5)	0	1 (2.9)	2 (3.3)	0	2 (6.5)
HSAL	*N* = 7	*N* = 1	*N* = 5	*N* = 6	*N* = 1	*N* = 3	*N* = 5	*N* = 1	*N* = 2	*N* = 4	*N* = 1	*N* = 2	*N* = 3	*N* = 0	*N* = 2
Median (range)	111.0 (18–193)	18.0 (18–18)	111.0 (38–193)	42.5 (0–151)	0.0 (0–0)	50.0 (6–133)	48.0 (0–89)	0.0 (0–0)	62.5 (48–77)	61.5 (0–109)	0.0 (0–0)	61.5 (30–93)	124.0 (26–137)	–	75.0 (26–124)

Numbers of good sleepers (PSQI< 5) and poor sleepers (PSQI≥5) do not add up to number of all patients due to missing PSQI baseline values

*PSQI* Pittsburgh Sleep Quality Index, *EDSS* Expanded Disability Status Scale, *RLS* restless legs syndrome, *MSFC* Multiple Sclerosis Functional Composite, *SD* standard deviation, *HSAL* Hamburg Pain Adjective List

The MS Functional Composite was lower in poor sleepers throughout the study and the EDSS score was higher at most visits (Table 3).

### Correlations of sleep quality and other comorbidities

There was a strong positive correlation between the PSQI and MFIS total scores at baseline and all follow-up visits, with correlation coefficients ranging from 0.62 to 0.71 (nominal *p* < 0.0001 at all time points; Table 4, Additional file 4). Moderate to strong positive correlations were also found between the PSQI and MFIS physical subscale (r_s_ = 0.58–0.67; nominal *p* < 0.0001 at all time points), MFIS cognitive subscale (r_s_ = 0.56–0.67; nominal p < 0.0001 at all time points), and MFIS psychological functioning subscale (r_s_ = 0.56–0.65; nominal *p* < 0.0001 at all time points; Table 4, Additional file 4).

**Table 4:** Correlations between the primary and secondary outcome variables

	Baseline visit						12-month visit						24-month visit					
	**PSQI (total score)**			MFIS (total score)			PSQI (total score)			MFIS (total score)			PSQI (total score)			MFIS (total score)		
	N	r_s_	p	N	r_s_	p	N	r_s_	p	N	r_s_	p	N	r_s_	p	N	r_s_	p
**PSQI (total score)**		–	–	106	**0.62**	<.0001		–	–	82	**0.68**	<.0001				41	**0.66**	<.0001
**MFIS(total score)**	106	**0.62**	<.0001		–	–	82	**0.68**	<.0001		–	–	41	**0.66**	<.0001			
**SF-36**																		
Physical component score (PCS)	100	−0.54	<.0001	113	**− 0.72**	<.0001	81	**−0.63**	<.0001	85	**−0.75**	<.0001	40	**−0.61**	<.0001	42	**−0.72**	<.0001
Mental component score (MCS)	100	−0.47	<.0001	113	**−0.68**	<.0001	81	−0.57	<.0001	85	**−0.81**	<.0001	40	**−0.78**	<.0001	42	**−0.78**	<.0001
**ESS score**	103	0.27	0.0049				82	0.55	<.0001				40	**0.49**	0.0011			
**HADS anxiety**	105	0.56	<.0001				81	0.51	<.0001				41	**0.53**	0.0002			
**HADS depression**	105	**0.60**	<.0001				81	0.44	<.0001				41	**0.55**	0.0001			
**HSAL score**	6	**0.93**	0.0045				5	**0.97**	0.0021				3	**1.00**	.			
**IRLSSG score**	4	**0.50**	0.5828				3	**0.50**	.				2	**1.00**	.			

Strong correlations are highlighted in **bold numbers**. *PSQI* Pittsburgh Sleep Quality Index, *MFIS* Modified Fatigue Impact Scale, *SF-36* Short Form-36, *ESS* Epworth Sleepiness Scale, *HADS* Hospital Anxiety and Depression Scale, *HSA*L Hamburg Pain Adjective List, *IRLSSG* International Restless Legs Symptom Study Group

Strong to moderate negative correlations at all visits were found between the PSQI total score and the SF-36 PCS (r_s_ = − 0.51−− 0.63; nominal *p* < 0.0001 at all time points) and the SF-36 MCS (r_s_ = − 0.47−− 0.78; nominal *p* < 0.0001 at all time points; Table 4, Additional file 4).

Weak to moderate positive correlations were found between the PSQI total score and ESS (r_s_ = 0.27–0.55; nominal *p* between 0.005 and < 0.0001), and between PSQI total score and HADS anxiety subscale (r_s_ = 0.51–0.56; nominal *p* between 0.0002 and < 0.0001; Table 4, Additional file 4). Moderate to strong positive correlations were found between the PSQI total score and HADS depression subscale (r_s_ = 0.44–0.60; nominal *p* between 0.0001 and < 0.0001; Table 4, Additional file 4).

**Fig. 2:**
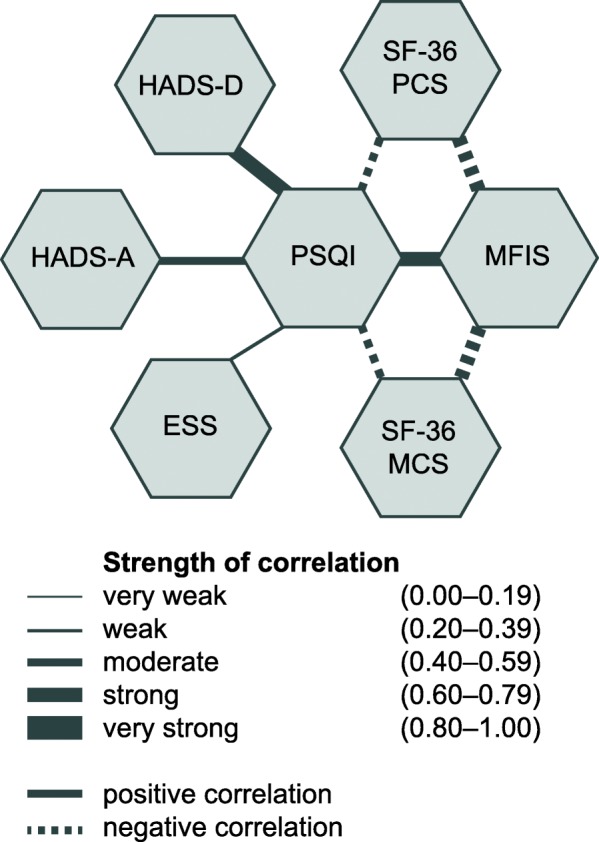
Direction and strength of correlations between questionnaire results for primary and secondary outcome variables at the beginning of the study. *PSQI* Pittsburgh Sleep Quality Index, *MFIS* Modified Fatigue Impact Scale, *SF-36* Short Form 36, *PCS* physical component score, *MCS* mental component score, *ESS* Epworth Sleepiness Scale, *HADS-D* Hospital Anxiety and Depression Scale Depression Subscale, *HADS-A* Hospital Anxiety and Depression Scale Anxiety Subscale

The strengths of correlations among all primary and secondary outcome measures are visualized in Fig. 2.

Further investigations using multivariable-adjusted linear regression analyses controlling for age, gender, EDSS score, and duration of disease supported the significant relationships seen in the correlation analysis (Additional file 5). An impact of duration of disease on PSQI scores was seen in most of these models. The influence of the questionnaire score was always the stronger one.

### Predictors of poor sleep quality

In univariate logistic regression analysis, poor sleep quality (PSQI≥5) at the one-year follow-up was associated with higher BMI (odds ratio [OR] 1.122, 95% confidence interval [CI] 1.004–1.254), poor sleep quality at baseline (OR 6.270, 95% CI 2.211–17.784), baseline ESS scores (OR 1.200, 95% CI 1.066–1.351), depression at baseline (OR 4.833, 95% CI 1.001–23.344), and anxiety at baseline (OR 3.741, 95% CI 1.217–11.338). Poor sleep quality at the two-year follow-up was predicted by age (OR 1.073, 95% CI 1.009–1.141), poor sleep quality at baseline (OR 4.500, 95% CI 1.06–19.111), and anxiety at baseline (OR 8.727, 95% CI 1.623–46.935).

In multivariate logistic regression using a stepwise selection procedure, baseline ESS values (OR 1.190, 95% CI 1.039–1.362) and poor sleep quality at baseline (OR 5.980, 95% CI 1.914–18.68) were identified as possible predictors for sleep quality at the one-year follow-up. No variable predicted sleep quality at the two-year follow-up.

## Discussion

In the BETASLEEP study, more than half of the patients reported poor sleep quality (PSQI≥5) at baseline, while the proportion was only 37.7% (95% CI 25.61–51.04%) after two years. Poor sleep quality was correlated with fatigue, low functional health status, and high scores of daytime sleepiness, depression, and anxiety.

The proportion of poor sleepers reported at the beginning of our study (55.5%) is comparable to that reported in other studies in Germany [14, 15], confirming the high prevalence of poor sleep among patients with MS. In a prospective study by Kotterba et al. among 73 patients with RRMS or CIS, the proportion of poor sleepers was ~ 50% [14]. In a recent cross-sectional study by Rupprecht et al. among 2062 MS patients irrespective of disease course poor sleep quality was present in 54 to 60% of patients [15]. This proportion is higher than what was recently reported in the general population. A study among 9284 people from a German community sample showed poor sleep quality in 36% of participants [16]. The smaller proportion of poor sleepers at the end of our study compared to the beginning may be due to the decreasing number of participants with evaluable PSQI questionnaire results over the course of the study. On the other hand a stable course of disease during interferon beta-1b may reduce fears concerning the development of the disease and improve sleep quality.

The cross-sectional study by Rupprecht et al. [15] further found that depression (96%), anxiety (88%), and fatigue (45%) were the most common comorbidities. In our study, depression was only present in 15.4 to 22.7% and anxiety was only present in 25.0 to 34.9% of patients. HADS-D scores in our study ranged from 3.92 to 4.91, and HADS-A scores ranged from 4.72 to 6.28. A large study among 4516 MS patients from the UK [17] found higher values for HADS-D (7.73) and HADS-A (8.03). In a German study by Kleiter et al. [18], values for HADS-D (3.7) and HADS-A (5.3) were slightly lower than in our study. The low average EDSS values in our study could be one explanation for a lower prevalence of depression and anxiety.

The study by Rupprecht et al. [15] identified anxiety and fatigue as predictors of poor sleep, while medication showed no effect. Furthermore, in the study by Kotterba et al. [14], poor sleep and fatigue were correlated. Our study confirmed the correlation of poor sleep and fatigue, as well as the association of poor sleep and anxiety. Both fatigue and poor sleep quality have repeatedly been shown to negatively affect quality of life in MS patients [4, 19, 20]. In the present study, fatigue and poor sleep were also associated with reduced quality of life assessed with the SF-36.

In contrast to poor sleep, excessive daytime sleepiness was only reported by between 26.4 and 36.4% of our patients. This finding is consistent with previous findings showing presence of excessive daytime sleepiness in around a quarter of MS patients [14].

MS treatment may influence sleep quality. Available results on the effects of interferon beta-1b on sleep quality are mixed. Some studies report negative effects [19, 21], others beneficial [22] or no effect on sleep quality [6]. In animals, it was shown that interferon type I receptors affect the sleep wake cycle [23]. In order to minimize potential differences in medication effects, only patients who had been treated with interferon beta-1b (Betaferon®) for less than six months and who had tolerated the treatment, were included in the study. Treatment tolerance was required in order to reduce the number of patients prematurely stopping the study.

In the present study, RLS was reported in 2.75 to 6.56% of patients. This proportion is much lower compared to other studies, where diagnosis of RLS was mostly based on questionnaires (prevalence of 14.4 to 57.5%; [2]) and standardized questionnaire-based interviews (prevalence of 32%; [18]). In contrast, in the present study, RLS was assessed by treating physicians based on their evaluation in routine clinical practice. When physicians diagnosed RLS in a patient, the severity was estimated with the IRLSSG. However, treating physicians might not have routinely asked for RLS symptoms. Thus, it is likely that RLS is underreported. The short duration of disease might have further contributed to the low prevalence of RLS in the present study. RLS increases with age in the general population and with disease duration and severity in MS (14). In the presented study patients are mildly impaired and in an early stage of the disease.

One of the advantages of our study is the prospective observational study design investigating sleep quality in German MS patients over two years, thus allowing a real-world picture to be drawn. Furthermore, key characteristics and results from questionnaires suggest that participants in the BETASLEEP study are comparable to other cohorts of patients with relapsing forms of MS with a similar functional health status [24] and a slightly lower level of depression and anxiety [17].

Limitations include the lack of a control group. The results therefore allow no conclusion regarding a possible treatment effect. However, the study was not designed to compare the effect of different medications on the course of sleep quality and fatigue, but rather to investigate sleep quality and fatigue under stable treatment conditions. The ideal situation would have been to prospectively investigate the natural course in untreated patients, which however is not possible due to ethical concerns. Further, obstructive sleep apnea was not excluded in patients. Given the observational study design reflecting real-world activities, such screening was not possible. Additional limitations include the low number of participants with RLS and chronic pain, precluding a reliable evaluation of the impact of these conditions on sleep quality and fatigue. Also, a considerable amount of patients was lost to follow-up. This might be due to the observational nature of the study, reflecting the real-world process in patient care. In addition, the patients were only mildly impaired and potentially observable changes may only occur over longer periods of time. Also we cannot draw any conclusion regarding the course of patients who are more severely affected. Finally, we used a forward selection procedure to identy potential predictors of poor sleep quality, which is prone to type I error. Given that the performed analyses are exploratory we wanted to make sure that we do not miss a potential predictor. This could have been the case with backward stepwise selection, for example, which sometimes drops variables that would be significant when added to the final reduced model.

## Conclusion

Taken together, our study confirms the high prevalence of poor sleep quality among patients with MS, which can also be seen in our cohort treated with interferon beta-1b over 2 years. Poor sleep quality was correlated with greater fatigue, lower functional health, and more depression and anxiety. The results highlight the importance of interventions targeted at improving sleep quality in patients with MS.

## Additional files

Additional file 1: Additional information on questionnaires, training of investigators and data collection. (PDF 62 kb)

Additional file 2: Flow chart of patients enrolled into the BETASLEEP study. (PDF 669 kb)

Additional file 3: Sleep quality during the course of the BETASLEEP study. (PDF 663 kb)

Additional file 4: Correlations between primary and secondary outcome variables. (PDF 153 kb)

Additional file 5: Multivariable-adjusted linear regression analysis for the influence of health status questionnaires on PSQI controlling for age, gender, EDSS, and duration of disease at baseline. (DOCX 17 kb)

## Abbreviations

BMI: Body mass index
CI: Confidence interval
CIS: Clinically isolated syndrome
DMD: Disease modifying drug
EDSS: Expanded disability status scale
ESS: Epworth Sleepiness Scale
HADS: Hospital Anxiety and Depression Scale
HSAL: Hamburg Pain Adjective List
IRLSSG: International RLS Study Group
MCS: Mental component score
MFIS: Modified fatigue impact scale
MS: Multiple sclerosis
OR: Odds ratio
PCS: Physical component score
PSQI: Pittsburgh Sleep Quality Index
RLS: Restless legs syndrome
RRMS: Relapsing remitting multiple sclerosis
SD: Standard deviation

## References

[1] Browne P, Chandraratna D, Angood C, Tremlett H, Baker C, Taylor BV (2014). Atlas of multiple sclerosis 2013: a growing global problem with widespread inequity. Neurology.

[2] Marrie RA, Reider N, Cohen J, Trojano M, Sorensen PS, Cutter G (2015). A systematic review of the incidence and prevalence of sleep disorders and seizure disorders in multiple sclerosis. Mult Scler.

[3] Barun B (2013). Pathophysiological background and clinical characteristics of sleep disorders in multiple sclerosis. Clin Neurol Neurosurg.

[4] Lobentanz IS, Asenbaum S, Vass K, Sauter C, Klosch G, Kollegger H (2004). Factors influencing quality of life in multiple sclerosis patients: disability, depressive mood, fatigue and sleep quality. Acta Neurol Scand.

[5] Fleming WE, Pollak CP (2005). Sleep disorders in multiple sclerosis. Semin Neurol.

[6] Neau JP, Paquereau J, Auche V, Mathis S, Godeneche G, Ciron J (2012). Sleep disorders and multiple sclerosis: a clinical and polysomnography study. Eur Neurol.

[7] Cameron MH, Peterson V, Boudreau EA, Downs A, Lovera J, Kim E (2014). Fatigue is associated with poor sleep in people with multiple sclerosis and cognitive impairment. Mult Scler Int.

[8] Krupp LB, Serafin DJ, Christodoulou C (2010). Multiple sclerosis-associated fatigue. Expert Rev Neurother.

[9] Veauthier C, Paul F (2014). Sleep disorders in multiple sclerosis and their relationship to fatigue. Sleep Med.

[10] Veauthier C, Radbruch H, Gaede G, Pfueller CF, Dorr J, Bellmann-Strobl J (2011). Fatigue in multiple sclerosis is closely related to sleep disorders: a polysomnographic cross-sectional study. Mult Scler.

[11] Cote I, Trojan DA, Kaminska M, Cardoso M, Benedetti A, Weiss D (2013). Impact of sleep disorder treatment on fatigue in multiple sclerosis. Mult Scler.

[12] Li Y, Munger KL, Batool-Anwar S, De Vito K, Ascherio A, Gao X (2012). Association of multiple sclerosis with restless legs syndrome and other sleep disorders in women. Neurology.

[13] Lanza G, Ferri R, Bella R, Ferini-Strambi L (2017). The impact of drugs for multiple sclerosis on sleep. Mult Scler.

[14] Kotterba S, Schwenkreis P, Schölzel W, Haltenhof C (2016). Fatigue and sleep problems in patients with relapsing-remitting multiple sclerosis (RRMS) under treatment with interferon β-1b. Klin Neurophysiol.

[15] Rupprecht S, Witte OW, Schwab M, for the SLEEP-MS Study Group. SLEEP-MS: Prevalence of Sleep Disturbances, Fatigue, Anxiety and Depression in Multiple Sclerosis. Presentation at the 90 Congress of the German Society of Neurology. Leipzig; 2017.

[16] Hinz A, Glaesmer H, Brahler E, Loffler M, Engel C, Enzenbach C, Hegerl U, Sander C (2017). Sleep quality in the general population: psychometric properties of the Pittsburgh sleep quality index, derived from a German community sample of 9284 people. Sleep Med.

[17] Jones KH, Jones PA, Middleton RM, Ford DV, Tuite-Dalton K, Lockhart-Jones H (2014). Physical disability, anxiety and depression in people with MS: an internet-based survey via the UK MS register. PLoS One.

[18] Kleiter I, Lang M, Jeske J, Norenberg C, Stollfuss B, Schurks M (2017). Adherence, satisfaction and functional health status among patients with multiple sclerosis using the BETACONNECT(R) autoinjector: a prospective observational cohort study. BMC Neurol.

[19] Boe Lunde HM, Aae TF, Indrevag W, Aarseth J, Bjorvatn B, Myhr KM (2012). Poor sleep in patients with multiple sclerosis. PLoS One.

[20] Amato MP, Ponziani G, Rossi F, Liedl CL, Stefanile C, Rossi L (2001). Quality of life in multiple sclerosis: the impact of depression, fatigue and disability. Mult Scler.

[21] Mendozzi L, Tronci F, Garegnani M, Pugnetti L (2009). Sleep disturbance and fatigue in mild relapsing remitting multiple sclerosis patients on chronic immunomodulant therapy: an actigraphic study. Mult Scler J.

[22] Pokryszko-Dragan A, Bilinska M, Gruszka E, Biel L, Kaminska K, Konieczna K (2013). Sleep disturbances in patients with multiple sclerosis. Neurological Sci.

[23] Bohnet SG, Traynor TR, Majde JA, Kacsoh B, Krueger JM (2004). Mice deficient in the interferon type I receptor have reduced REM sleep and altered hypothalamic hypocretin, prolactin and 2′,5′-oligoadenylate synthetase expression. Brain Res.

[24] Baumstarck K, Butzkueven H, Fernández O, Flachenecker P, Stecchi S, Idiman E (2013). Responsiveness of the multiple sclerosis international quality of life questionnaire to disability change: a longitudinal study. Health Qual Life Outcomes.

