# Supplementary material for: Sleep quality, daytime sleepiness, fatigue, and quality of life in patients with multiple sclerosis treated with interferon beta-1b: results from a prospective observational cohort study
Source: BMC Neurol. 2018 Aug 24;18:123. doi: 10.1186/s12883-018-1113-5 (PMC6107945; doi:10.1186/s12883-018-1113-5)
Supplement: Supplementary file 1 — Additional information on questionnaires, training of investigators and data collection. (PDF 62 kb) [file 12883_2018_1113_MOESM1_ESM.pdf]

Sleep quality, daytime sleepiness, fatigue, and quality of life in patients with multiple sclerosis treated with interferon beta-1b: results from a prospective observational cohort study

Sylvia Kotterba, MD; Thomas Neusser, PhD, Christiane Norenberg, MSc, Patrick Bussfeld, MD[4], Thomas Glaser, PhD, Martin Dörner, PhD, Markus Schürks, MD, MSc

Additional file 1 - Methods

Questionnaires

The PSQI [1] assesses sleep quality and disturbances retrospectively over a four-week period. Nineteen individual items rated on a four point scale (0–3) generate seven component scores: subjective sleep quality, sleep latency, sleep duration, habitual sleep efficiency, sleep disturbances, use of sleep-inducing medication, and daytime dysfunction. The sum of the component scores yields one global score, which can range from 0 to 21. The higher the global score, the lower the sleep quality. A cut-off value of five was used to differentiate good from poor sleepers [2, 3].

The MFIS [4] consists of 21 items selected from the Fatigue Impact Scale [5], a multidimensional scale developed to assess the perceived impact of fatigue on a variety of daily activities. The items are rated on a five point scale (0–4) and make up three subscales (physical, cognitive, and psychosocial). A total MFIS score can be calculated by adding the scores of the subscales. All items are scaled so that higher scores indicate a greater impact of fatigue on a patient’s activities.

The SF-36 [6], a health survey with 36 items rated on a six-point scale (1–6), includes one multi-item scale that assesses eight health concepts: 1) limitations in physical activities because of health problems; 2) limitations in social activities because of physical or emotional problems; 3) limitations in usual role activities because of physical health problems; 4) bodily pain; 5) general mental health (psychological distress and well-being); 6) limitations in usual role activities because of emotional problems; 7) vitality (energy and fatigue); and 8) general health perceptions. Each scale is directly transformed into a scale ranging from 0 to 100.

The ESS [7] is an eight-question survey providing a measure of a patient’s general level of daytime sleepiness, or his/her average sleep propensity in daily life. Each item is rated on a four-point scale (0–3). The total ESS score is the sum of eight item scores and can range between 0 and 24.The higher the score, the higher the person’s level of daytime sleepiness.

The HADS [8] was used to record depression and anxiety. It is a fourteen-item scale with seven items relating to anxiety and seven relating to depression. Each item on the questionnaire is scored from 0–3, and total scores for anxiety or depression can range from 0 to 21. The cut-off point for depression or anxiety is 8/21.

The HSAL [9] determines the multidimensional sensation of pain in adult patients with acute or chronic pain. Using 37 pain adjectives, affective and sensory pain quality are recorded on a seven point scale. The quantification of pain is based on the following four dimensions: pain suffering, pain intensity, fear of pain, and rhythm of pain.

The IRLSSG rating scale [10] is a self-administered tool consisting of 10 questions evaluating the severity of symptoms in people suffering from RLS. Each question has a five-level response scale (0–4), with the total score ranging from 0 to 40.

The MSFC [11] comprises quantitative functional measures of three key clinical dimensions of MS: leg function/ambulation assessed with a timed 25-foot walk test, arm/hand function assessed with the nine-hole peg test, and cognitive function assessed with the Paced Auditory Serial Addition Test. Scores on component measures are converted to standard scores (z-scores), which are averaged to form a single MSFC score.

The EDSS [12] is a widely used scale to assess the grade of disability in patients with MS. The neurological functions of the patients are evaluated in eight separate functional systems from which the grade of disability will be derived on a scale from 0 (no disability) to 10 (death).

Training of investigators

Study investigators were obliged to attend an online training presentation prior to enrolment of patients. The training provided detailed information about the aims and course of the study as well as the process of electronic data documentation.

Data collection

All data were entered into electronic case report forms (eCRFs) and saved in an electronic data capture system (EDC system). At the baseline visit, the investigators documented demographic data (age, gender, race, body mass index [BMI]), employment status, educational level, and medical history. Medical history comprised the date of the first clinical event suggestive of MS, the date of initial diagnosis including McDonald or Poser criteria, the number of further demyelinating events/relapses, and concomitant diseases of special interest (sleep disorders, RLS, depression or anxiety, fatigue, chronic pain including headache). Betaferon® administration and concomitant medication were documented at all visits. The disease course (number of further demyelinating events/relapses with date of onset) including changes in disease course (change from RRMS to secondary progressive MS and number of further demyelinating events/relapses since the previous visit with date of onset), and adverse events were documented at all follow-up visits. The EDSS was documented at all visits, while the MSFC was documented at baseline and at the one-year and two-year follow-ups. All outcome variables were documented at all study visits.

Patient-reported outcomes were documented using questionnaires (PSQI, ESS, SF-36, MFIS, HADS, IRLSSG rating scale, HSAL; see section “Outcome variables”), collected by the

investigator and sent to the contract research organization (CRO), where the data were double-entered into the eCRF.

Patients with available data at baseline as well as at least one post-baseline visit were included in the full analysis set (FAS).

References

1. Buysse DJ, Reynolds CF, 3rd, Monk TH, Berman SR, Kupfer DJ. The Pittsburgh Sleep Quality Index: a new instrument for psychiatric practice and research. Psychiatry research. 1989;28(2):193-213.

2. Boe Lunde HM, Aae TF, Indrevag W, Aarseth J, Bjorvatn B, Myhr KM, et al. Poor sleep in patients with multiple sclerosis. PloS one. 2012;7(11):e49996.

3. Kotterba S, Schwenkreis P, Schölzel W, Haltenhof C. [Fatigue and Sleep Problems in Patients with Relapsing-remitting Multiple Sclerosis (RRMS) under Treatment with Interferon β-1b]. Klin Neurophysiol. 2016;47(03):136-41.

4. Fisk JD, Pontefract A, Ritvo PG, Archibald CJ, Murray TJ. The impact of fatigue on patients with multiple sclerosis. The Canadian journal of neurological sciences Le journal canadien des sciences neurologiques. 1994;21(1):9-14.

5. Fisk JD, Ritvo PG, Ross L, Haase DA, Marrie TJ, Schlech WF. Measuring the functional impact of fatigue: initial validation of the fatigue impact scale. Clinical infectious diseases : an official publication of the Infectious Diseases Society of America. 1994;18 Suppl 1:S79-83.

6. Ware JE, Jr., Sherbourne CD. The MOS 36-item short-form health survey (SF-36). I. Conceptual framework and item selection. Medical care. 1992;30(6):473-83.

7. Johns MW. A new method for measuring daytime sleepiness: the Epworth sleepiness scale. Sleep. 1991;14(6):540-5.

8. Zigmond AS, Snaith RP. The hospital anxiety and depression scale. Acta psychiatrica Scandinavica. 1983;67(6):361-70.

9. Hoppe F. Hamburger Schmerz-Adjektiv-Liste (HSAL). Manual. Weinheim: Beltz; 1991.

10. Walters AS, LeBrocq C, Dhar A, Hening W, Rosen R, Allen RP, et al. Validation of the International Restless Legs Syndrome Study Group rating scale for restless legs syndrome. Sleep medicine. 2003;4(2):121-32.

11. Fischer JS, Rudick RA, Cutter GR, Reingold SC. The Multiple Sclerosis Functional Composite Measure (MSFC): an integrated approach to MS clinical outcome assessment. National MS Society Clinical Outcomes Assessment Task Force. Multiple sclerosis (Houndmills, Basingstoke, England). 1999;5(4):244-50.

12. Kurtzke JF. Rating neurologic impairment in multiple sclerosis: an expanded disability status scale (EDSS). Neurology. 1983;33(11):1444-52.
