# Supplementary figures and images for: Sleep quality, daytime sleepiness, fatigue, and quality of life in patients with multiple sclerosis treated with interferon beta-1b: results from a prospective observational cohort study
Source: BMC Neurol. 2018 Aug 24;18:123. doi: 10.1186/s12883-018-1113-5 (PMC6107945; doi:10.1186/s12883-018-1113-5)

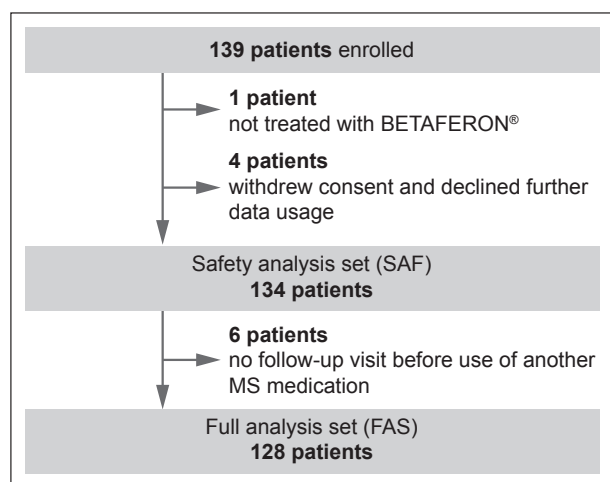

**Additional file 2:** Flow chart of patients enrolled into the BETASLEEP study.

Supplement: Supplementary file 2 — Flow chart of patients enrolled into the BETASLEEP study. (PDF 669 kb) [file 12883_2018_1113_MOESM2_ESM.pdf]
