# Supplementary material for: Sleep quality, daytime sleepiness, fatigue, and quality of life in patients with multiple sclerosis treated with interferon beta-1b: results from a prospective observational cohort study
Source: BMC Neurol. 2018 Aug 24;18:123. doi: 10.1186/s12883-018-1113-5 (PMC6107945; doi:10.1186/s12883-018-1113-5)
Supplement: Supplementary file 3 — Sleep quality during the course of the BETASLEEP study. (PDF 663 kb) [file 12883_2018_1113_MOESM3_ESM.pdf]

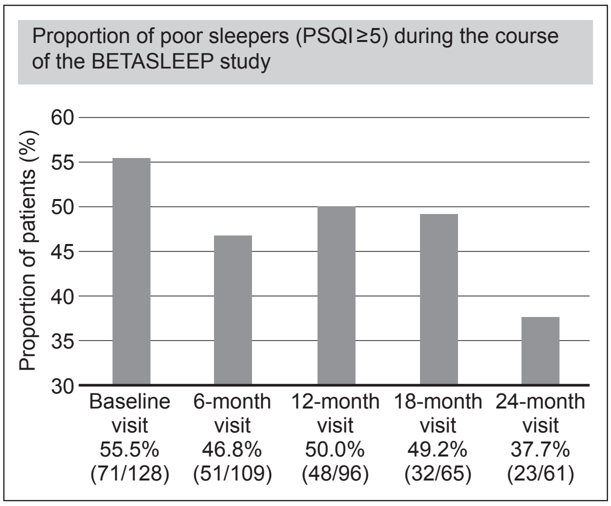

Additional file 3: Sleep quality during the course of the BETASLEEP study.
PSQI, Pittsburg Sleep Quality Index.
