# Supplementary material for: Sleep quality, daytime sleepiness, fatigue, and quality of life in patients with multiple sclerosis treated with interferon beta-1b: results from a prospective observational cohort study
Source: BMC Neurol. 2018 Aug 24;18:123. doi: 10.1186/s12883-018-1113-5 (PMC6107945; doi:10.1186/s12883-018-1113-5)
Supplement: Supplementary file 4 — Correlations between primary and secondary outcome variables. (PDF 153 kb) [file 12883_2018_1113_MOESM4_ESM.pdf]

Additional file 4: Correlations between the primary and secondary outcome variables.

	Baseline visit						6-month visit						12-month visit						18-month visit						24-month visit					
	PSQI (total score)			**MFIS (total score)**			**PSQI (total score)**			**MFIS (total score)**			**PSQI (total score)**			**MFIS (total score)**			**PSQI (total score)**			**MFIS (total score)**			**PSQI (total score)**			**MFIS (total score)**		
	N	r_s	p	N	r_s	p	N	r_s	p	N	r_s	p	N	r_s	p	N	r_s	p	N	r_s	p	N	r_s	p	N	r_s	p	N	r_s	p
**PSQI**																														
Total score		-	-	106	**0.62**	<.0001		-	-	90	**0.63**	<.0001		-	-	82	**0.68**	<.0001				51	**0.71**	<.0001				41	**0.66**	<.0001
Duration of sleep		-	-	117	0.20	0.0297		-	-	95	0.10	0.3122		-	-	86	0.19	0.0799				52	0.13	0.3487				43	0.05	0.7277
Sleep disturbance		-	-	112	0.51	<.0001		-	-	100	0.52	<.0001		-	-	85	**0.60**	<.0001				51	0.53	<.0001				42	0.37	0.0159
Sleep latency		-	-	119	0.41	<.0001		-	-	96	0.38	0.0001		-	-	85	0.43	<.0001				52	0.47	0.0004				43	0.42	0.0041
Day dysfunction		-	-	121	**0.67**	<.0001		-	-	102	**0.74**	<.0001		-	-	87	**0.75**	<.0001				53	**0.82**	<.0001				43	**0.85**	<.0001
Sleep efficiency		-	-	115	0.27	0.0033		-	-	94	0.29	0.0043		-	-	85	0.44	<.0001				52	0.41	0.0025				43	0.26	0.0883
Overall sleep quality		-	-	119	0.45	<.0001		-	-	102	0.53	<.0001		-	-	87	0.51	<.0001				52	**0.60**	<.0001				42	0.41	0.0061
Need medication		-	-	120	0.38	<.0001		-	-	100	0.34	0.0005		-	-	87	0.37	0.0004				52	0.42	0.0017				42	0.37	0.0147
**MFIS**																														
Total score	106	**0.62**	<.0001		-	-	90	**0.63**	<.0001		-	-	82	**0.68**	<.0001		-	-	51	**0.71**	<.0001				41	**0.66**	<.0001			
Physical	106	**0.61**	<.0001		-	-	90	0.58	<.0001		-	-	82	**0.66**	<.0001		-	-	51	**0.67**	<.0001				41	**0.65**	<.0001			
Cognitive	106	0.56	<.0001		-	-	90	**0.60**	<.0001		-	-	82	**0.64**	<.0001		-	-	51	**0.67**	<.0001				41	**0.61**	<.0001			
Psychosocial functioning	106	**0.60**	<.0001		-	-	90	0.59	<.0001		-	-	82	0.56	<.0001		-	-	51	**0.65**	<.0001				41	**0.60**	<.0001			
**SF-36**																														
Physical components summary (PCS)	100	-0.54	<.0001	113	**-0.72**	<.0001	84	-0.51	<.0001	96	**-0.77**	<.0001	81	**-0.63**	<.0001	85	**-0.75**	<.0001	49	**-0.62**	<.0001	51	**-0.75**	<.0001	40	**-0.61**	<.0001	42	**-0.72**	<.0001
Mental components summary (MCS)	100	-0.47	<.0001	113	**-0.68**	<.0001	84	**-0.62**	<.0001	96	**-0.76**	<.0001	81	-0.57	<.0001	85	**-0.81**	<.0001	49	-0.59	<.0001	51	**-0.69**	<.0001	40	**-0.78**	<.0001	42	**-0.78**	<.0001
Vitality	104	-0.38	<.0001	119	**-0.65**	<.0001	89	-0.43	<.0001	101	**-0.70**	<.0001	82	-0.58	<.0001	86	**-0.69**	<.0001	49	-0.51	0.0002	51	**-0.60**	<.0001	40	-0.55	0.0001	42	**-0.72**	<.0001
Physical functioning	105	**-0.63**	<.0001	119	-0.59	<.0001	87	-0.57	<.0001	99	**-0.69**	<.0001	82	-0.56	<.0001	87	**-0.67**	<.0001	50	**-0.70**	<.0001	52	**-0.69**	<.0001	40	-0.56	0.0001	42	-0.58	<.0001
Bodily pain subscale	104	-0.50	<.0001	117	**-0.78**	<.0001	88	-0.54	<.0001	100	**-0.79**	<.0001	82	**-0.66**	<.0001	87	**-0.82**	<.0001	50	**-0.65**	<.0001	52	**-0.78**	<.0001	40	**-0.66**	<.0001	42	**-0.77**	<.0001
General health perceptions	103	-0.40	<.0001	117	-0.47	<.0001	88	-0.51	<.0001	100	**-0.60**	<.0001	82	-0.51	<.0001	87	**-0.67**	<.0001	50	-0.51	0.0001	52	-0.48	0.0002	40	**-0.63**	<.0001	42	**-0.60**	<.0001
Physical role functioning	104	-0.46	<.0001	119	**-0.78**	<.0001	89	-0.43	<.0001	101	**-0.72**	<.0001	81	**-0.61**	<.0001	86	**-0.70**	<.0001	50	-0.44	0.0013	52	**-0.64**	<.0001	40	-0.53	0.0003	42	**-0.72**	<.0001
Emotional role functioning	104	-0.34	0.0004	119	-0.54	<.0001	88	-0.57	<.0001	100	**-0.63**	<.0001	81	-0.48	<.0001	86	**-0.60**	<.0001	50	-0.45	0.0008	52	-0.51	<.0001	40	**-0.65**	<.0001	42	-0.57	<.0001
Social role functioning	103	-0.51	<.0001	117	**-0.61**	<.0001	87	-0.49	<.0001	99	**-0.70**	<.0001	82	-0.51	<.0001	87	**-0.77**	<.0001	50	-0.56	<.0001	52	**-0.67**	<.0001	40	**-0.60**	<.0001	42	**-0.79**	<.0001
Mental health	104	-0.54	<.0001	118	**-0.61**	<.0001	88	-0.51	<.0001	100	**-0.68**	<.0001	82	-0.55	<.0001	87	**-0.77**	<.0001	50	-0.53	<.0001	52	**-0.68**	<.0001	40	**-0.70**	<.0001	42	**-0.73**	<.0001
**ESS score**	103	0.27	0.0049										82	0.55	<.0001										40	**0.49**	0.0011			
**HADS anxiety**	105	0.56	<.0001										81	0.51	<.0001										41	**0.53**	0.0002			
**HADS depression**	105	**0.60**	<.0001										81	0.44	<.0001										41	**0.55**	0.0001			
**HSAL suffering from pain**	6	**0.93**	0.0045										6	**0.81**	0.0499										3	**1.00**	.			
**HSAL anxiety pain**	6	**0.90**	0.0112										6	**0.90**	0.0112										3	**1.00**	.			
**HSAL pain intensity**	6	**0.94**	0.0025										5	**0.89**	0.0428										3	**0.50**	.			
**HSAL pain rhythm**	6	**0.93**	0.0045										6	0.44	0.4120										3	**1.00**	.			
**HSAL affective**	6	**0.84**	0.0341										6	**0.81**	0.0499										3	**1.00**	.			
**HSAL sensory**	6	**0.93**	0.0045										5	**0.97**	0.0021										3	**0.50**	.			
**HSAL score**	6	**0.93**	0.0045										5	**0.97**	0.0021										3	**1.00**	.			
**IRLSSG symptom**	4	0.32	0.7433										3	0.50	.										2	**1.00**	.			
**IRLSSG impact**	4	**0.95**	0.0690										3	0.50	.										3	**0.00**	.			
**IRLSSG score**	4	0.50	0.5828										3	0.50	.										2	**1.00**	.			

Strong correlations are highlighted in **bold** numbers. *PSQI* Pittsburgh Sleep Quality Index; *MFIS* Modified Fatigue Impact Scale; *SF-36* Short Form-36; *ESS* Epworth Sleepiness Scale; *HADS* Hospital Anxiety and Depression Scale; *HSAL* Hamburg Pain Adjective List; *IRLSSG* International Restless Legs Symptom Study Group
