# Supplementary material for: Sleep quality, daytime sleepiness, fatigue, and quality of life in patients with multiple sclerosis treated with interferon beta-1b: results from a prospective observational cohort study
Source: BMC Neurol. 2018 Aug 24;18:123. doi: 10.1186/s12883-018-1113-5 (PMC6107945; doi:10.1186/s12883-018-1113-5)
Supplement: Supplementary file 5 — Multivariable-adjusted linear regression analysis for the influence of health status questionnaires on PSQI controlling for age, gender, EDSS, and duration of disease at baseline. (DOCX 17 kb) [file 12883_2018_1113_MOESM5_ESM.docx]

**Additional file 5: Multivariable-adjusted linear regression analyses for the influence of health status questionnaires on PSQI controlling for age, gender, EDSS, duration of disease at baseline**

| **Independent variables** | **Regression model for** | | | | | | | | | | | |
| --- | --- | --- | --- | --- | --- | --- | --- | --- | --- | --- | --- | --- |
|  | **MFIS (total score)** | | | **SF-36 Physical component score (PCS)** | | | **SF-36 Mental component score (MCS)** | | | **ESS score** | | |
|  | N | est | p-value | N | est | p-value | N | est | p-value | N | est | p-value |
| **Questionnaire score** | 106 | 0.13 | **< .0001** | 100 | -0.23 | **< .0001** | 100 | -0.15 | **< .0001** | 106 | 0.22 | **0.016** |
| **Age** | 106 | 0.00 | 0.9377 | 100 | -0.07 | **0.0468** | 100 | 0.00 | 0.9731 | 106 | 0.01 | 0.8260 |
| **Gender (female vs male)** | 106 | 0.92 | 0.2212 | 100 | 0.57 | 0.4556 | 100 | 0.61 | 0.4593 | 106 | 1.66 | 0.0726 |
| **EDSS score** | 106 | -0.31 | 0.3412 | 100 | -0.33 | 0.3246 | 100 | -0.19 | 0.5859 | 106 | 0.05 | 0.8897 |
| **Duration of disease** | 106 | 0.01 | **0.0228** | 100 | 0.01 | 0.1111 | 100 | 0.02 | **0.0091** | 106 | 0.01 | **0.0466** |

**Additional file 5 continued**

| **Independent variable** | **Regression model for** | | | | | |
| --- | --- | --- | --- | --- | --- | --- |
|  | **HADS anxiety** | | | **HADS depression** | | |
|  | N | est | p-value | N | est | p-value |
| **Questionnaire score** | 105 | 0.59 | **< .0001** | 105 | 0.64 | **< .0001** |
| **Age** | 105 | 0.01 | 0.7188 | 105 | -0.02 | 0.5883 |
| **Gender (female vs male)** | 105 | 0.69 | 0.3809 | 105 | 1.11 | 0.1325 |
| **EDSS score** | 105 | -0.24 | 0.4789 | 105 | -0.22 | 0.4905 |
| **Duration of disease** | 105 | 0.01 | **0.0114** | 105 | 0.01 | **0.0382** |

*PSQI* Pittsburgh Sleep Quality Index, *MFIS* Modified Fatigue Impact Scale, *SF-36* Short Form-36, *ESS* Epworth Sleepiness Scale, *HADS* Hospital Anxiety and Depression Scale, *est* estimate. P-values <0.05 are indicated in bold.
